# Automatic Mobile Health Arrhythmia Monitoring for the Detection of Atrial Fibrillation: Prospective Feasibility, Accuracy, and User Experience Study

**DOI:** 10.2196/29933

**Published:** 2021-10-22

**Authors:** Onni E Santala, Jari Halonen, Susanna Martikainen, Helena Jäntti, Tuomas T Rissanen, Mika P Tarvainen, Tomi P Laitinen, Tiina M Laitinen, Eemu-Samuli Väliaho, Juha E K Hartikainen, Tero J Martikainen, Jukka A Lipponen

**Affiliations:** 1 School of Medicine Faculty of Health Sciences University of Eastern Finland Kuopio Finland; 2 Doctoral School Faculty of Health Sciences University of Eastern Finland Kuopio Finland; 3 Heart Center Kuopio University Hospital Kuopio Finland; 4 Department of Health and Social Management University of Eastern Finland Kuopio Finland; 5 Center for Prehospital Emergency Care Kuopio University Hospital Kuopio Finland; 6 Heart Center North Karelia Central Hospital Joensuu Finland; 7 Department of Applied Physics University of Eastern Finland Kuopio Finland; 8 Department of Clinical Physiology and Nuclear Medicine Kuopio University Hospital Kuopio Finland; 9 Department of Emergency Care Kuopio University Hospital Kuopio Finland

**Keywords:** atrial fibrillation, ECG, algorithm, stroke, mHealth, user experience, Awario analysis Service, Suunto Movesense, cardiology, digital health, mobile health, wearable device, heart belt, arrhythmia monitor, heart monitor

## Abstract

**Background:**

Atrial fibrillation (AF) is the most common tachyarrhythmia and associated with a risk of stroke. The detection and diagnosis of AF represent a major clinical challenge due to AF’s asymptomatic and intermittent nature. Novel consumer-grade mobile health (mHealth) products with automatic arrhythmia detection could be an option for long-term electrocardiogram (ECG)-based rhythm monitoring and AF detection.

**Objective:**

We evaluated the feasibility and accuracy of a wearable automated mHealth arrhythmia monitoring system, including a consumer-grade, single-lead heart rate belt ECG device (heart belt), a mobile phone application, and a cloud service with an artificial intelligence (AI) arrhythmia detection algorithm for AF detection. The specific aim of this proof-of-concept study was to test the feasibility of the entire sequence of operations from ECG recording to AI arrhythmia analysis and ultimately to final AF detection.

**Methods:**

Patients (n=159) with an AF (n=73) or sinus rhythm (n=86) were recruited from the emergency department. A single-lead heart belt ECG was recorded for 24 hours. Simultaneously registered 3-lead ECGs (Holter) served as the gold standard for the final rhythm diagnostics and as a reference device in a user experience survey with patients over 65 years of age (high-risk group).

**Results:**

The heart belt provided a high-quality ECG recording for visual interpretation resulting in 100% accuracy, sensitivity, and specificity of AF detection. The accuracy of AF detection with the automatic AI arrhythmia detection from the heart belt ECG recording was also high (97.5%), and the sensitivity and specificity were 100% and 95.4%, respectively. The correlation between the automatic estimated AF burden and the true AF burden from Holter recording was >0.99 with a mean burden error of 0.05 (SD 0.26) hours. The heart belt demonstrated good user experience and did not significantly interfere with the patient’s daily activities. The patients preferred the heart belt over Holter ECG for rhythm monitoring (85/110, 77% heart belt vs 77/109, 71% Holter, *P*=.049).

**Conclusions:**

A consumer-grade, single-lead ECG heart belt provided good-quality ECG for rhythm diagnosis. The mHealth arrhythmia monitoring system, consisting of heart-belt ECG, a mobile phone application, and an automated AF detection achieved AF detection with high accuracy, sensitivity, and specificity. In addition, the mHealth arrhythmia monitoring system showed good user experience.

**Trial Registration:**

ClinicalTrials.gov NCT03507335; https://clinicaltrials.gov/ct2/show/NCT03507335

## Introduction

Atrial fibrillation (AF) is the most common tachyarrhythmia diagnosed in clinical practice. The aging of the global population is expected to increase the prevalence of AF by 2.3-fold by 2030 as compared with 2010 [1]. The most serious complication of AF is embolic stroke. The stroke risk can be reduced by as much as 60% with oral anticoagulation therapy in high-risk AF patients [2-6]. Evidently, there is a need to develop new approaches to diagnose AF in patients who would benefit from anticoagulation.

According to the current recommendations from the European Society of Cardiology (ESC), an electrocardiography (ECG) documentation interpreted by a physician is required to establish the diagnosis of AF [7]. AF screening recommendations include opportunistic or systematic screening in patients ≥65 years of age or with other characteristics suggesting an increased risk of stroke [7]. The clinical challenge is that AF is often paroxysmal or asymptomatic. Thus, it remains often undiagnosed when using traditional 12-lead ECG or Holter recordings [8,9]. Even the widely available patient-triggered mobile health (mHealth) products have not been able to resolve this challenge in asymptomatic patients [10]. User experience also is particularly important in the development of mHealth products, as these technologies can be too technical and complex for elderly people (ie, those who would benefit most from AF screening and anticoagulation therapy) [11]. In the development of medical technology, user involvement has positive effects, such as increased awareness of users’ needs and experiences, better design, and clearer interfaces, as well as improved functionality, usability, and quality [12].

In this proof-of-concept study, we evaluated the feasibility and accuracy of the entire sequence of operations from ECG recording to artificial intelligence (AI) arrhythmia detection and the diagnosis of AF. This novel mHealth arrhythmia self-monitoring system includes a commonly available consumer-grade, single-lead heart rate belt ECG device (heart belt), a mobile phone application, and a cloud service with an AI arrhythmia detection algorithm.

The specific aims of the study were to (1) evaluate the feasibility and quality of a single lead heart belt for ECG recording, (2) determine the accuracy of the heart belt ECG in AF diagnosis, (3) assess the accuracy of the AI arrhythmia detection algorithm for AF screening, and (4) evaluate the user experience with the heart belt in a subgroup of patients >65 years of age (high-risk group).

## Methods

### Study Design

This study was part of a larger study entity, Atrial Fibrillation Detection: 24 Hour Study (AFIB24h), in which several different measurement techniques for detecting AF were studied. The study was performed as a single-center study at Kuopio University Hospital. The local ethics committee approved the study protocol (July 23, 2017), and the study was registered in the ClinicalTrials.gov database (NCT03507335).

### Recruitment

The inclusion criteria were AF or sinus rhythm (SR) based on a 12-lead resting ECG recorded during admission to the hospital. The exclusion criteria were (1) estimated stay in the hospital <24 hours, (2) BMI ≥35 kg/m^2^, (3) left bundle branch block (LBBB) or right bundle branch block (RBBB), (4) implanted cardiac pacemaker, and (5) a medical condition requiring immediate treatment.

**Figure 1:**
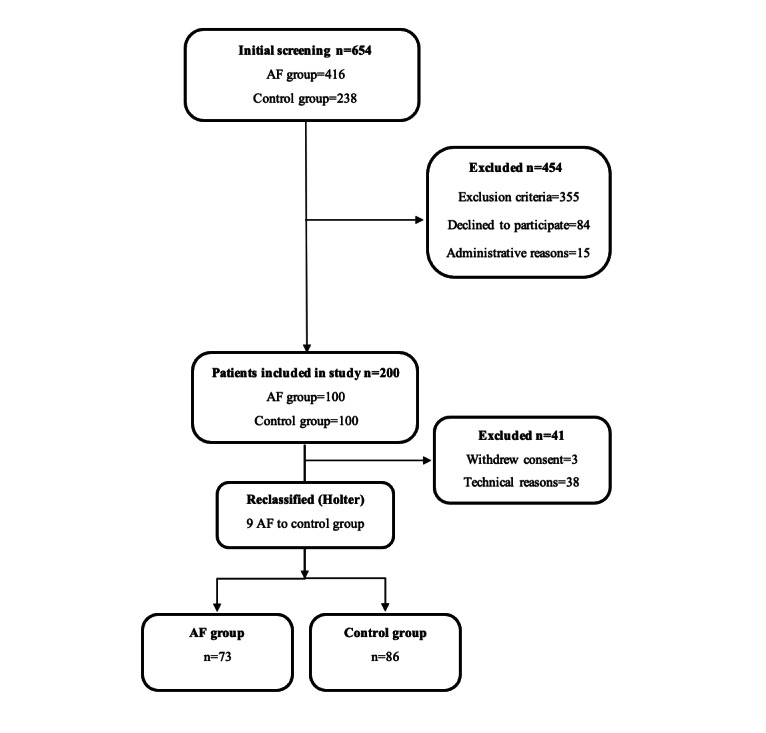
Study flow chart. AF: atrial fibrillation.

A total of 654 patients were screened in the emergency department between April 2018 and December 2019. In the initial screening, 454 patients were excluded for the reasons summarized in Figure 1. Of the remaining 200 patients, 100 patients with AF were assigned to the AF group, and 100 patients with SR were assigned to the control group. However, a further 41 patients were excluded: 38 patients due to technical reasons and 3 patients who withdrew their consent. In addition, the rhythm of some patients had converted from the time of 12-lead ECG recording prior to study measurements. Consequently, the final rhythm classification made from Holter ECG recording reclassified 9 patients from the AF group to the control group. Thus, the final study population consisted of 159 patients, of whom 73 were in the AF group and 86 were in the control group. The clinical characteristics of the patients were collected using a standardized data collection protocol and confirmed or complemented from the medical records. All participants provided written informed consent to participate in the study. In addition, the performance of the AI arrhythmia detection algorithm for identifying short AF episodes was tested using 173 ECG recordings from 4 public AF-detection datasets (Multimedia Appendix 1).

### ECG Recordings

**Figure 2:**
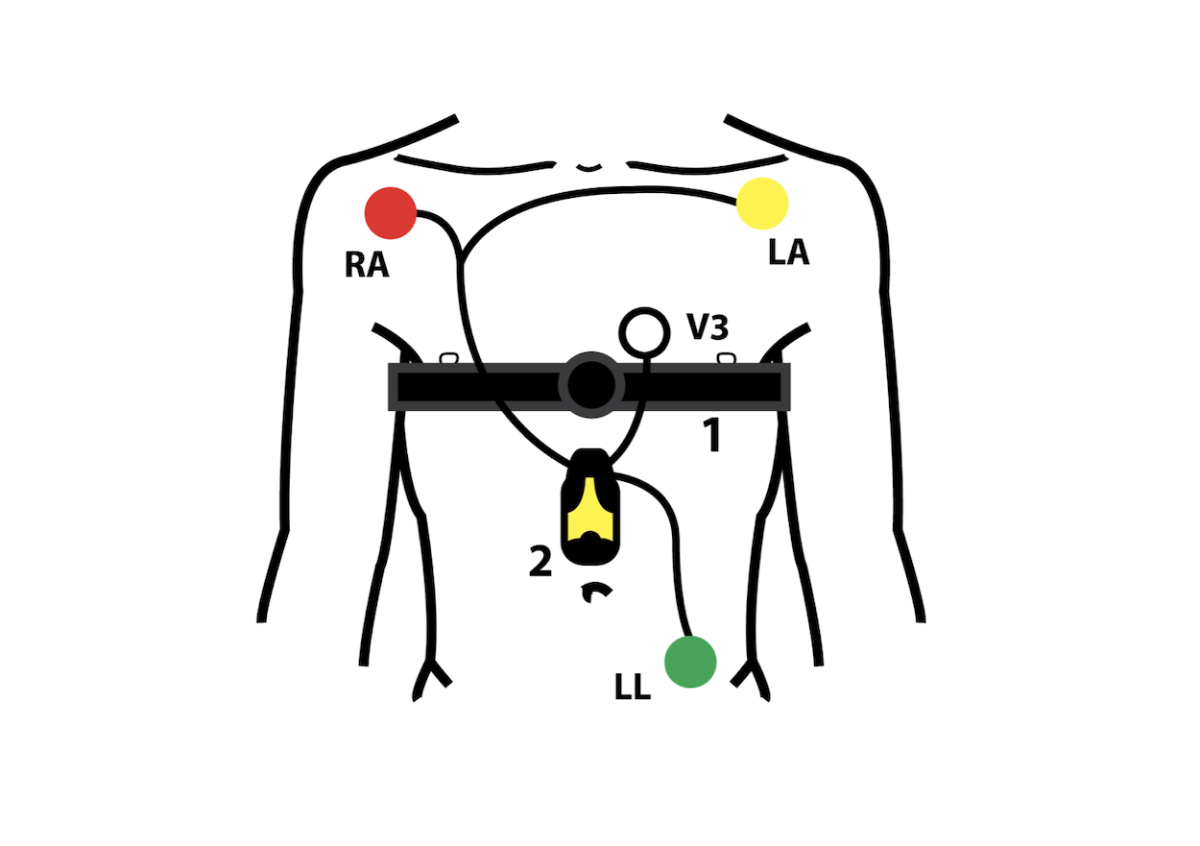
Electrocardiogram (ECG) recordings using a (1) single-lead heart belt ECG recording and (2) 3-lead Holter ECG recording. LA: left arm; LL: left limb; RA: right arm; V3: V3 lead of the 12-lead ECG.

**Figure 3:**
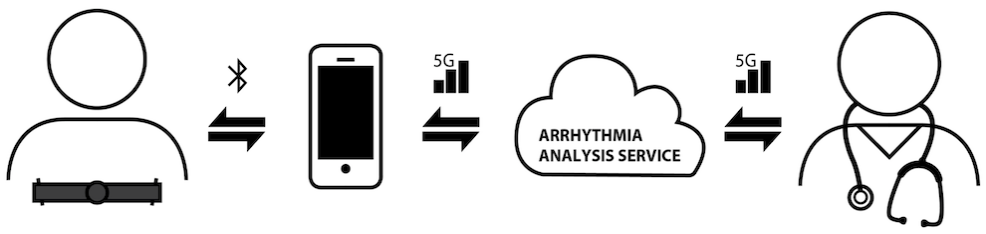
Schematic presentation of the heart belt electrocardiogram (ECG)-based automatic arrhythmia detection.

A consumer-grade, single-lead heart belt (Suunto Movesense, Suunto, Vantaa, Finland) was attached to the patient’s chest approximately 2 cm below the lower end of the sternum (Figure 2). The heart belt ECG data were transferred via Bluetooth connection to a mobile phone from where the data were transmitted to a cloud service for both visual and automatic analyses (Figure 3). A heart belt ECG was recorded for 24 hours. A simultaneously registered 3-lead Holter ECG recording (Faros 360, Bittium, Oulu, Finland) was used as the gold standard for rhythm classification (Figure 2).

### ECG Analysis

The heart belt ECG and Holter recordings were both analyzed using Medilog Darwin Professional V2.8.1 software (Schiller Global, Baar, Switzerland). The ECG recordings were reviewed in a random order independently by 4 investigators blinded to the 12-lead ECG and classified as either an AF or non-AF rhythm. Subsequently, commercial AI arrhythmia analysis software (Awario, Heart2Save, Kuopio, Finland) was used for automatic AF screening from the heart belt ECG recordings. The AI arrhythmia detection algorithm classified the heart belt ECG data into SR, AF, or noninterpretable. The accuracy of the visual and automatic rhythm classifications from the heart belt ECG recording was further assessed by comparing it with the gold standard Holter ECG recording. The rhythm analyses were performed from recordings in which both the heart belt ECG and Holter ECG recordings were interpretable.

### Heart Belt User Experience

The patients were asked to complete a user experience questionnaire developed for this study at the end of the ECG registration. Patients evaluated their heart belt and Holter experience separately, answering the following questions for each device: how you rank the device (comfortable=1, reasonably comfortable=2, neutral=3, slightly uncomfortable=4, uncomfortable=5); did the device interfere with sleep, eating, toileting, or movement (Yes or No); and would you be willing to use the device at home for rhythm monitoring (Yes or No). The user experience was analyzed in a subgroup of patients >65 years of age (ie, in those patients in whom the AF screening is recommended by the ESC guidelines)**.**

### Statistical Analysis

The AF and control groups were compared using *t* tests for continuous variables and χ^2^ tests or Fisher exact tests for dichotomous variables. The following parameters were used to quantify the performance of (1) detecting AF per patient (subject-based) and (2) total accumulated AF duration across all patients (time-based) from heart belt ECG recordings visually and with an AI arrhythmia detection algorithm: accuracy, sensitivity, specificity, negative predictive value (NPV), and positive predictive value (PPV). The absolute difference between the AF burden derived from the heart belt and the Holter was described using the mean AF burden error (time). The AF burden determined by the heart belt was compared with the reference AF burden from the Holter using a Bland-Altman plot [13]. In the survey assessing user experience, the users’ opinions of the heart belt and Holter were compared using Wilcoxon signed rank tests and McNemar tests. All significance tests were two-tailed, and *P*≤.05 was considered statistically significant. The data were analyzed using SPSS version 25.

## Results

### Clinical Characteristics

In comparison with the control group, AF patients were older (mean 77, SD 10 years vs mean 68, SD 16 years; *P*<.001), presented more often with a history of paroxysmal AF (*P*<.001) or congestive heart failure (*P*<.001), and were more often on anticoagulation (*P*<.001), digoxin (*P*=.045), and beta-blocker (*P*=.02) therapy (Table 1). Furthermore, the AF patients also reported more often the presence of palpitations (*P*=.02) and respiratory distress (*P*=.001).

**Table 1:** Patient demographics.

Characteristics			Control group (n=86)		AF^a^ group (n=73)		Significance (2-sided)
Age (years), mean (SD)			68 (16)		77 (10)		<.001
BMI (kg/m^2^), mean (SD)			26 (4)		27 (4)		.31
Male gender, n (%)			34 (40)		38 (52)		.11
**Medical history, n (%)**							
	Earlier AF episode	17 (20)		58 (80)		<.001	
	Coronary heart disease	22 (26)		22 (30)		.52	
	Diabetes mellitus	19 (22)		17 (23)		.86	
	Hypertension	51 (59)		53 (73)		.08	
	Congestive heart failure	11 (13)		34 (47)		<.001	
	Previous heart surgery	5 (6)		10 (14)		.09	
**Medication, n (%)**							
	Anticoagulation therapy	22 (26)		61 (84)		<.001	
	Beta-blocker	40 (47)		48 (66)		.02	
	Digoxin	4 (5)		10 (14)		.045	
	Anti-arrhythmic medication	0 (0)		2 (3)		.21	
**Symptoms prior to hospital admission, n (%)**							
	Decrease in general condition	48 (56)		43 (59)		.70	
	Fatigue	47 (55)		44 (60)		.48	
	Palpitations	22 (26)		32 (44)		.02	
	Respiratory distress	24 (28)		39 (53)		.001	
	Chest pain	16 (19)		13 (18)		.90	

^a^AF: atrial fibrillation.

### Quality of the Heart Belt ECG Data

**Figure 4:**
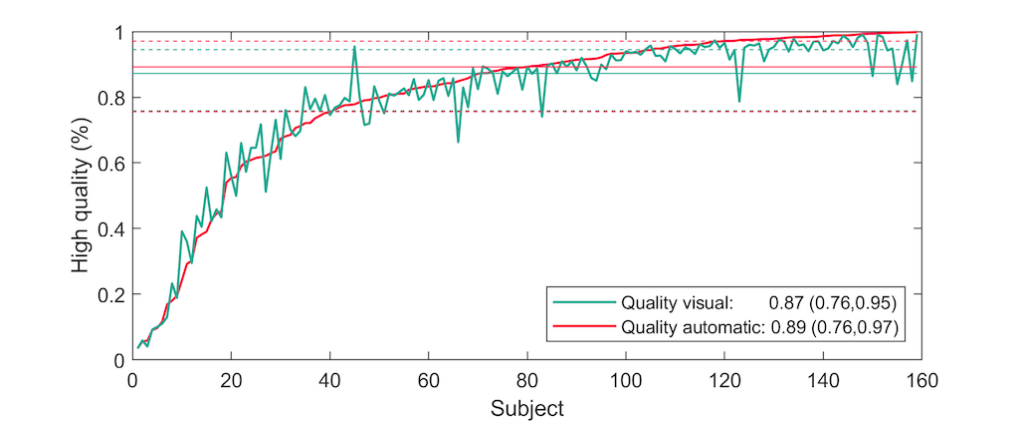
Percentage of interpretable electrocardiograms (ECGs) in individual subject recordings, which are sorted using an automatic quality value.

**Figure 5:**
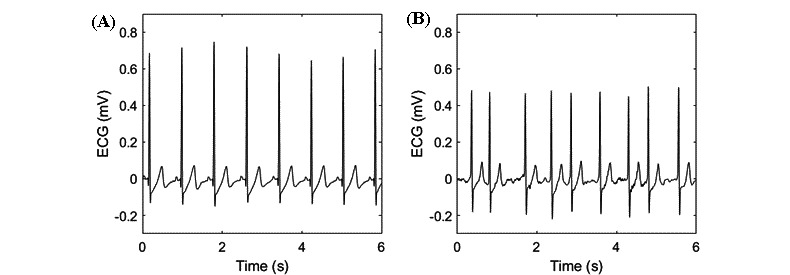
Examples of heart belt electrocardiogram (ECG) recordings for (A) sinus rhythm and (B) atrial fibrillation.

In the analysis of heart belt ECG data (all subjects), 2707 hours (2707.44/3416.81, 79.24%) of visual analysis and 2748 hours (2747.73/3416.81, 80.42%) of automatic analysis were deemed interpretable. Based on the visual assessment, 1226 hours (1225.59/1566.45, 78.24%) of the AF group recordings and 1482 hours (1481.85/1850.40, 80.08%) of the control group recordings were judged as being interpretable. Correspondingly, 1224 hours (1223.91/1566.39, 78.14%) of the AF recordings and 1524 hours (1523.82/1850.40, 82.35%) of the control group recordings were interpretable when analyzed with the AI arrhythmia detection algorithm. The subject-based median for visually interpretable data was 87% (25th percentile=76%; 75th percentile=95%), very similar to the automatic analysis, (median 89%; 25th percentile=76%; 75th percentile=97%; Figure 4). The accuracy of visual and automated rhythm analyses was evaluated from the ECG data deemed interpretable in both heart belt and Holter ECG recordings (2655.72 hours). Representative examples of heart belt ECG measurements are presented in Figure 5.

### Accuracy of Visual Assessment From Heart Belt ECG

The quality of the ECG signal from the mHealth system was tested by classifying the heart belt ECG recordings visually into AF and non-AF rhythms. In the subject-based analysis, all the patients with AF were correctly identified, and correspondingly, none of the subjects with SR were given a false AF diagnosis (Table 2). In the time-based analysis, the accuracy, sensitivity, and specificity of diagnosing AF from the heart belt ECG recordings were all >99.9%. Correspondingly, the PPV and NPV of detecting the presence or absence of AF were both >99.9%.

**Table 2:** Subject- and time-based atrial fibrillation (AF) detection accuracy, sensitivity, specificity, positive predictive value (PPV), and negative predictive value (NPV) based on visual and automatic artificial intelligence (AI) arrhythmia algorithms.

Type of algorithm	Accuracy, %	Sensitivity, %	Specificity, %	PPV, %	NPV, %
Visual subject-based	100	100	100	100	100
Visual time-based	>99.9	>99.9	>99.9	>99.9	>99.9
Algorithm subject-based	97.5	100	95.4	94.8	100
Algorithm time-based	99.2	98.5	99.7	99.6	>99.9

### Accuracy of the AI Arrhythmia Detection Algorithm From the Heart Belt ECG

**Figure 6:**
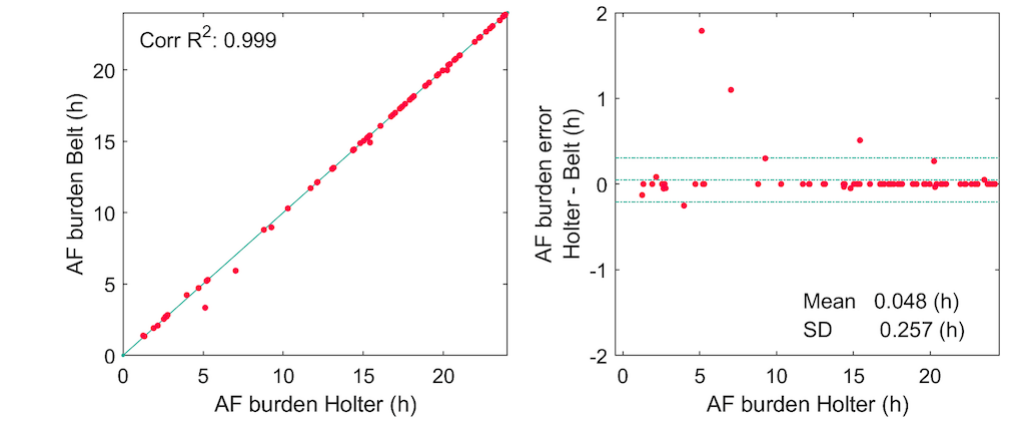
Correlation between atrial fibrillation (AF) burden estimated by the artificial intelligence (AI) arrhythmia detection algorithm and the reference AF burden from (A) Holter recording and (B) Bland Altman plot of the AF burden estimate.

The AI arrhythmia detection algorithm detected AF correctly in all the patients in the AF group and suggested the presence of AF in 4 patients in the SR group (false-positive AF detection), resulting in a subject-based accuracy of 97.5%, sensitivity of 100%, specificity of 95.4%, PPV of 94.8%, and NPV of 100% (Table 2). The time-based accuracy, sensitivity, and specificity values for the AI arrhythmia detection algorithm for identifying AF were 99.2%, 98.5%, and 99.7%, respectively, and the PPV and NPV for detecting the presence or absence of AF were 99.6% and >99.9%, respectively (Table 2). The correlation between the true and automatically estimated AF burden from AF patients was >0.99, and the mean burden error was 0.05 (SD 0.26) hours (Figure 6).

The supplementary analyses performed for 4 freely available ECG datasets including different arrhythmias found that the sensitivity of the AI arrhythmia detection algorithm to detect short AF episodes was moderate. The AI arrhythmia detection algorithm detected 83.43% (2421/2902) of all AF episodes with a duration of >30 seconds, but the sensitivity increased significantly with the duration of an AF episode. The AI arrhythmia detection algorithm detected 95.10% (641/674) of AF episodes lasting >5 minutes and 98.49% (327/332) of episodes with a duration >15 minutes (Figure S1 in Multimedia Appendix 1). The overall time-based accuracy, sensitivity, and specificity for AF detection were high, at 97.8%, 97.1%, and 98.4%, respectively (Table S1 in Multimedia Appendix 1). The correlation between the reference AF burden and the estimated AF burden was >0.99, and the mean burden error was 0.05 (SD 0.82) hours (Figure S2 in Multimedia Appendix 1).

### User Experience With the Heart Belt

A total of 112 patients over 65 years of age filled in the user experience survey. Using a scale of 1 to 5 (1=comfortable and 5=uncomfortable), the patients rated the heart belt as a median of 3 (25th percentile=2; 75th percentile=4; Table 3). In terms of discomfort, 9 (9/112, 8.0%) patients reported that the heart belt device interfered with their sleep, 3 (3/112, 2.7%) with eating, 4 (4/112, 3.6%) with toileting, and 4 (4/112, 3.6%) with normal movement. Finally, 85 (85/110, 77.3%) of the patients reported that they would be willing to use the heart belt device, slightly more than the 77 (77/109, 70.6%) who stated that they would wear the Holter device at home for rhythm monitoring (*P*=.049; Table 3).

**Table 3:** User experience with the heart belt and Holter devices.

Question		Holter	Heart belt	Significance (2-sided)
How would you rank the device^a^, median (25th percentile, 75th percentile)		3 (2,3)^b^	3 (2,4)^c^	.06^b^
**Device interference, n (%)**				
	Device interfered with sleep	9 (8.0)^d^	9 (8.0)^d^	1.0^e^
	Device interfered with eating	1 (0.9)^d^	3 (2.7)^d^	.63^e^
	Device interfered with toileting	4 (3.6)^d^	4 (3.6)^d^	1.0^e^
	Device interfered with movement	1 (0.9)^d^	4 (3.6)^d^	.40^e^
**Experience with device usability, n (%)**				
	I would use the device at home for rhythm monitoring	77 (70.6)^f^	85 (77.3)^c^	.049^f^

^a^Comfortable=1, reasonably comfortable=2, neutral=3, slightly uncomfortable=4, uncomfortable=5.

^b^n=108.

^c^n=110.

^d^n=112.

^e^n=111.

^f^n=109.

^g^n=107.

## Discussion

### Principal Findings

We demonstrated that a novel mHealth arrhythmia monitoring system using a consumer-grade heart belt ECG device, a mobile phone application, and an automated AI arrhythmia analysis was both feasible and accurate for 24-hour ECG monitoring and rhythm diagnostics. The heart belt provided a high-quality ECG signal for visual evaluation, achieving an AF diagnostic accuracy of 100%. In addition, the AI arrhythmia detection algorithm identified AF patients with a sensitivity of 100% and a specificity of 95.4% (4 false positives).

In our study, 80% of the heart belt ECG recordings were of sufficient quality to permit visual and automatic rhythm diagnostics. The proportion of analyzable data using the heart belt operating with dry electrodes was comparable with results obtained in earlier studies using adhesive-coated wet electrodes or shirt-type ECG recording devices [14-16]. In these publications, the amount of analyzable data varied from 92% to 99% of the total wear time, which ranged from 48 hours to several days.

AF diagnosis requires confirmation by a physician [7]. Thus, the ECG signal provided by the mHealth system needs to be of high quality. In previous studies, the sensitivity of AF detection by health care professionals using mHealth ECG recordings has ranged from 73% to 100%, with specificity from 84% to 100% [17-20]. In our study, the accuracy of AF diagnosis was superior to previous studies. In the visual assessment, all AF patients were identified, and no patient with SR was misdiagnosed as having AF. Thus, the mHealth monitoring system described here represents a useful tool for identification of AF.

Visual analysis of long-term ECG recording for AF screening is very time and manpower consuming. Thus, there is an unmet need for automatic AF detection. In previous studies, the sensitivity of identifying AF using arrhythmia detection algorithms from mHealth ECG recording has ranged from 87% to 99%, with a specificity from 80% to 97% [21-26]. In our study, when using the AI ​​arrhythmia detection algorithm, the AF detection sensitivity (100%) was superior, and the specificity (95.4%) was comparable or even superior to those in previous studies. Only 4 of 86 patients had a false AF alarm, and, in most cases, this was due to frequent (>10,000 per 24 hours) supraventricular or ventricular extrasystoles. Although the accuracy of current “state-of-the-art” AF detection algorithms is high, the presence of false positives (approximately 2-5/100) indicates that AF diagnosis needs to be confirmed by a physician. Nonetheless, the benefits of automated AI arrhythmia detection in ambulatory screening are evident; they can (1) exclude poor quality data, (2) detect AF patients with high sensitivity and specificity, and (3) exclude >90% of patients who do not require medical attention. In addition to AF diagnosis, the frequency and duration of an AF episode as well as the AF burden are associated with the risk of stroke [27-31]. We found that the AF burden estimated by the AI arrhythmia detection algorithm correlated almost perfectly with the true AF burden (*r*>0.99).

Previous studies have reported poor compliance with lead-based, long-term ECG monitoring devices, such as Holter devices, mobile telemetry devices, and event monitors, because of their difficulty in use; interference with the patient’s work, travel, or lifestyle; and skin irritation [32-35]. In contrast, novel mHealth methods have been more comfortable, causing less interference with daily living; therefore, participants have preferred the new mHealth ECG devices instead of the traditional Holter for rhythm monitoring [36,37]. In our study, the heart belt user experience was found to be significantly better than the Holter ECG in a subgroup of elderly patients (over 65 years of age). Indeed, a higher proportion (77.3% vs 70.6%) of elderly patients preferred the heart belt to the Holter device, the gold standard of rhythm monitoring. Patient-reported discomfort caused by the heart belt was very low. The advantage of heart belts over traditional measurement methods is that they have been designed to allow freedom of movement and users find them easy to use. It should be mentioned here that the Holter device used in this study was very small, weighing only 18 grams.

Several new photoplethysmography and ECG-based wearable mHealth technologies, such as smartphones and watches, have been studied for AF detection [18-20,38-41]. These wearable technologies could provide a practical and cost-effective solution for AF detection and AF burden assessment [7]. According to a recent survey [42], there is consensus on recommending mHealth wearable devices or apps as an alternative to traditional methods such as Holter monitoring for detecting AF in symptomatic patients or post stroke or TIA. However, health care professionals do not feel that health care systems are ready for mass consumer-initiated AF screening with these techniques, as there still appears to be a need to better define suitable screening population and an appropriate management pathway for consumers with positive results [42]. From the perspective of health care professionals, the presented approach with the heart belt monitoring has the potential to be a small change from traditional Holter and therefore to be widely accepted. Our study suggests that the presented mHealth monitoring technology enables long-term AF screening and can be considered as being user-friendly. An AI ​​arrhythmia monitoring system can warn the patient of a possible AF, store the ECG from the AF episode, and send it to the physician for a final rhythm confirmation. In addition to the AF diagnosis, the system could provide an estimate of the AF burden and help in the initiation of appropriate treatment for stroke prevention and rhythm control.

### Study Limitations

We acknowledge some limitations in our study. First, morbid obesity could degrade the signal quality and thus, produce more failed measurements. In addition, in cases with RBBB and LBBB, the presence of 2 broad R-peak QRS complexes could increase the variation of the R-R interval, which could result in false AF detection by the automatic algorithm. For these reasons, these patients were excluded, and further studies in these subgroups will be needed. Second, the study was conducted in a proof-of-concept style to test the feasibility of the entire sequence of operations, from ECG recording to automatic AF analysis. Examining a greater number of patients was not possible due to technical aspects related to telecommunication links to the server as well as some application problems with this proof-of-concept system. In addition, poor electrode contacts and incorrect placement of the heart belt caused some noninterpretable periods of recording. In addition, our mHealth system for AF monitoring should be studied in an out-of-hospital setting to assess the signal quality, the accuracy of AF detection, and AF burden estimate as well as the overall usability.

### Conclusions

A consumer-grade, single-lead ECG heart belt provided good-quality ECG for rhythm diagnosis. The mHealth arrhythmia monitoring system, consisting of heart-belt ECG, a mobile phone application, and automated AF detection achieved AF detection with high accuracy, sensitivity, and specificity. In addition, the mHealth arrhythmia monitoring system showed good user experience.

## Appendix

Multimedia Appendix 1 Supplementary material A.

## Abbreviations

AF: atrial fibrillation
AI: artificial intelligence
ECG: electrocardiogram
ESC: European Society of Cardiology
LBBB: left bundle branch block
mHealth: mobile health
NPV: negative predictive value
PPV: positive predictive value
RBBB: right bundle branch block
SR: sinus rhythm

## References

[1] Colilla S, Crow A, Petkun W, Singer DE, Simon T, Liu X (2013). Estimates of current and future incidence and prevalence of atrial fibrillation in the U.S. adult population. Am J Cardiol.

[2] Kishore A, Vail A, Majid A, Dawson J, Lees KR, Tyrrell PJ, Smith CJ (2014). Detection of atrial fibrillation after ischemic stroke or transient ischemic attack: a systematic review and meta-analysis. Stroke.

[3] Henriksson KM, Farahmand B, Åsberg S, Edvardsson N, Terént A (2012). Comparison of cardiovascular risk factors and survival in patients with ischemic or hemorrhagic stroke. Int J Stroke.

[4] Grond M, Jauss M, Hamann G, Stark E, Veltkamp R, Nabavi D, Horn M, Weimar C, Köhrmann M, Wachter R, Rosin L, Kirchhof P (2013). Improved detection of silent atrial fibrillation using 72-hour Holter ECG in patients with ischemic stroke: a prospective multicenter cohort study. Stroke.

[5] Hart RG, Pearce LA, Aguilar MI (2007). Meta-analysis: antithrombotic therapy to prevent stroke in patients who have nonvalvular atrial fibrillation. Ann Intern Med.

[6] Ruff CT, Giugliano RP, Braunwald E, Hoffman EB, Deenadayalu N, Ezekowitz MD, Camm AJ, Weitz JI, Lewis BS, Parkhomenko A, Yamashita T, Antman EM (2014). Comparison of the efficacy and safety of new oral anticoagulants with warfarin in patients with atrial fibrillation: a meta-analysis of randomised trials. Lancet.

[7] Hindricks G, Potpara T, Dagres N, Arbelo E, Bax JJ, Blomström-Lundqvist C, Boriani G, Castella M, Dan G, Dilaveris PE, Fauchier L, Filippatos G, Kalman JM, La Meir M, Lane DA, Lebeau J, Lettino M, Lip GYH, Pinto FJ, Thomas GN, Valgimigli M, Van Gelder IC, Van Putte BP, Watkins CL, ESC Scientific Document Group (2021). 2020 ESC Guidelines for the diagnosis and management of atrial fibrillation developed in collaboration with the European Association for Cardio-Thoracic Surgery (EACTS): The Task Force for the diagnosis and management of atrial fibrillation of the European Society of Cardiology (ESC) Developed with the special contribution of the European Heart Rhythm Association (EHRA) of the ESC. Eur Heart J.

[8] Kirchhof P, Bax J, Blomstrom-Lundquist C, Calkins H, Camm AJ, Cappato R, Cosio F, Crijns H, Diener H, Goette A, Israel CW, Kuck K, Lip GYH, Nattel S, Page RL, Ravens U, Schotten U, Steinbeck G, Vardas P, Waldo A, Wegscheider K, Willems S, Breithardt G (2009). Early and comprehensive management of atrial fibrillation: executive summary of the proceedings from the 2nd AFNET-EHRA consensus conference 'research perspectives in AF'. Eur Heart J.

[9] Healey JS, Alings M, Ha A, Leong-Sit P, Birnie DH, de Graaf JJ, Freericks M, Verma A, Wang J, Leong D, Dokainish H, Philippon F, Barake W, McIntyre WF, Simek K, Hill MD, Mehta SR, Carlson M, Smeele F, Pandey AS, Connolly SJ, ASSERT-II Investigators (2017). Subclinical Atrial Fibrillation in Older Patients. Circulation.

[10] Diederichsen SZ, Haugan KJ, Kronborg C, Graff C, Højberg S, Køber L, Krieger D, Holst AG, Nielsen JB, Brandes A, Svendsen JH (2020). Comprehensive Evaluation of Rhythm Monitoring Strategies in Screening for Atrial Fibrillation: Insights From Patients at Risk Monitored Long Term With an Implantable Loop Recorder. Circulation.

[11] Zapata BC, Fernández-Alemán JL, Idri A, Toval A (2015). Empirical studies on usability of mHealth apps: a systematic literature review. J Med Syst.

[12] Shah SGS, Robinson I, AlShawi S (2009). Developing medical device technologies from users' perspectives: a theoretical framework for involving users in the development process. Int J Technol Assess Health Care.

[13] Bland JM, Altman DG (1986). Statistical methods for assessing agreement between two methods of clinical measurement. Lancet.

[14] Turakhia MP, Hoang DD, Zimetbaum P, Miller JD, Froelicher VF, Kumar UN, Xu X, Yang F, Heidenreich PA (2013). Diagnostic utility of a novel leadless arrhythmia monitoring device. Am J Cardiol.

[15] Fukuma N, Hasumi E, Fujiu K, Waki K, Toyooka T, Komuro I, Ohe K (2019). Feasibility of a T-Shirt-Type Wearable Electrocardiography Monitor for Detection of Covert Atrial Fibrillation in Young Healthy Adults. Sci Rep.

[16] Solomon MD, Yang J, Sung SH, Livingston ML, Sarlas G, Lenane JC, Go AS (2016). Incidence and timing of potentially high-risk arrhythmias detected through long term continuous ambulatory electrocardiographic monitoring. BMC Cardiovasc Disord.

[17] Desteghe L, Raymaekers Z, Lutin M, Vijgen J, Dilling-Boer D, Koopman P, Schurmans J, Vanduynhoven P, Dendale P, Heidbuchel H (2017). Performance of handheld electrocardiogram devices to detect atrial fibrillation in a cardiology and geriatric ward setting. Europace.

[18] Tarakji KG, Wazni OM, Callahan T, Kanj M, Hakim AH, Wolski K, Wilkoff BL, Saliba W, Lindsay BD (2015). Using a novel wireless system for monitoring patients after the atrial fibrillation ablation procedure: the iTransmit study. Heart Rhythm.

[19] Lau JK, Lowres N, Neubeck L, Brieger DB, Sy RW, Galloway CD, Albert DE, Freedman SB (2013). iPhone ECG application for community screening to detect silent atrial fibrillation: a novel technology to prevent stroke. Int J Cardiol.

[20] Himmelreich JC, Karregat EP, Lucassen WA, van Weert HC, de Groot JR, Handoko ML, Nijveldt R, Harskamp RE (2019). Diagnostic Accuracy of a Smartphone-Operated, Single-Lead Electrocardiography Device for Detection of Rhythm and Conduction Abnormalities in Primary Care. Ann Fam Med.

[21] Haeberlin A, Roten L, Schilling M, Scarcia F, Niederhauser T, Vogel R, Fuhrer J, Tanner H (2014). Software-based detection of atrial fibrillation in long-term ECGs. Heart Rhythm.

[22] Lian J, Wang L, Muessig D (2011). A simple method to detect atrial fibrillation using RR intervals. Am J Cardiol.

[23] Duverney D, Gaspoz J, Pichot V, Roche F, Brion R, Antoniadis A, Barthélémy JC (2002). High accuracy of automatic detection of atrial fibrillation using wavelet transform of heart rate intervals. Pacing Clin Electrophysiol.

[24] Tateno K, Glass L (2001). Automatic detection of atrial fibrillation using the coefficient of variation and density histograms of RR and ΔRR intervals. Med. Biol. Eng. Comput.

[25] Hindricks G, Pokushalov E, Urban L, Taborsky M, Kuck K, Lebedev D, Rieger G, Pürerfellner H, XPECT Trial Investigators (2010). Performance of a new leadless implantable cardiac monitor in detecting and quantifying atrial fibrillation: Results of the XPECT trial. Circ Arrhythm Electrophysiol.

[26] Giebel GD, Gissel C (2019). Accuracy of mHealth Devices for Atrial Fibrillation Screening: Systematic Review. JMIR Mhealth Uhealth.

[27] Chen LY, Chung MK, Allen LA, Ezekowitz M, Furie KL, McCabe P, Noseworthy PA, Perez MV, Turakhia MP, American Heart Association Council on Clinical Cardiology, Council on CardiovascularStroke Nursing; Council on Quality of Care and Outcomes Research, Stroke Council (2018). Atrial Fibrillation Burden: Moving Beyond Atrial Fibrillation as a Binary Entity: A Scientific Statement From the American Heart Association. Circulation.

[28] Boriani G, Glotzer TV, Santini M, West TM, De Melis M, Sepsi M, Gasparini M, Lewalter T, Camm JA, Singer DE (2014). Device-detected atrial fibrillation and risk for stroke: an analysis of >10,000 patients from the SOS AF project (Stroke preventiOn Strategies based on Atrial Fibrillation information from implanted devices). Eur Heart J.

[29] Senoo K, Lip GYH, Lane DA, Büller HR, Kotecha D (2015). Residual Risk of Stroke and Death in Anticoagulated Patients According to the Type of Atrial Fibrillation: AMADEUS Trial. Stroke.

[30] Vanassche T, Lauw MN, Eikelboom JW, Healey JS, Hart RG, Alings M, Avezum A, Díaz R, Hohnloser SH, Lewis BS, Shestakovska O, Wang J, Connolly SJ (2015). Risk of ischaemic stroke according to pattern of atrial fibrillation: analysis of 6563 aspirin-treated patients in ACTIVE-A and AVERROES. Eur Heart J.

[31] Banerjee A, Taillandier S, Olesen JB, Lane DA, Lallemand B, Lip GYH, Fauchier L (2013). Pattern of atrial fibrillation and risk of outcomes: the Loire Valley Atrial Fibrillation Project. Int J Cardiol.

[32] Roten L, Schilling M, Häberlin A, Seiler J, Schwick NG, Fuhrer J, Delacrétaz E, Tanner H (2012). Is 7-day event triggered ECG recording equivalent to 7-day Holter ECG recording for atrial fibrillation screening?. Heart.

[33] Ackermans PAJ, Solosko TA, Spencer EC, Gehman SE, Nammi K, Engel J, Russell JK (2012). A user-friendly integrated monitor-adhesive patch for long-term ambulatory electrocardiogram monitoring. J Electrocardiol.

[34] Rothman SA, Laughlin JC, Seltzer J, Walia JS, Baman RI, Siouffi SY, Sangrigoli RM, Kowey PR (2007). The diagnosis of cardiac arrhythmias: a prospective multi-center randomized study comparing mobile cardiac outpatient telemetry versus standard loop event monitoring. J Cardiovasc Electrophysiol.

[35] Vasamreddy CR, Dalal D, Dong J, Cheng A, Spragg D, Lamiy SZ, Meininger G, Henrikson CA, Marine JE, Berger R, Calkins H (2006). Symptomatic and asymptomatic atrial fibrillation in patients undergoing radiofrequency catheter ablation. J Cardiovasc Electrophysiol.

[36] Smith WM, Riddell F, Madon M, Gleva MJ (2017). Comparison of diagnostic value using a small, single channel, P-wave centric sternal ECG monitoring patch with a standard 3-lead Holter system over 24 hours. Am Heart J.

[37] Barrett PM, Komatireddy R, Haaser S, Topol S, Sheard J, Encinas J, Fought AJ, Topol EJ (2014). Comparison of 24-hour Holter monitoring with 14-day novel adhesive patch electrocardiographic monitoring. Am J Med.

[38] Chan P, Wong C, Poh YC, Pun L, Leung WW, Wong Y, Wong MM, Poh M, Chu DW, Siu C (2016). Diagnostic Performance of a Smartphone-Based Photoplethysmographic Application for Atrial Fibrillation Screening in a Primary Care Setting. J Am Heart Assoc.

[39] Bumgarner JM, Lambert CT, Hussein AA, Cantillon DJ, Baranowski B, Wolski K, Lindsay BD, Wazni OM, Tarakji KG (2018). Smartwatch Algorithm for Automated Detection of Atrial Fibrillation. J Am Coll Cardiol.

[40] Rozen G, Vaid J, Hosseini SM, Kaadan MI, Rafael A, Roka A, Poh YC, Poh M, Heist EK, Ruskin JN (2018). Diagnostic Accuracy of a Novel Mobile Phone Application for the Detection and Monitoring of Atrial Fibrillation. Am J Cardiol.

[41] Orchard J, Lowres N, Freedman SB, Ladak L, Lee W, Zwar N, Peiris D, Kamaladasa Y, Li J, Neubeck L (2016). Screening for atrial fibrillation during influenza vaccinations by primary care nurses using a smartphone electrocardiograph (iECG): A feasibility study. Eur J Prev Cardiol.

[42] Boriani G, Schnabel RB, Healey JS, Lopes RD, Verbiest-van Gurp N, Lobban T, Camm JA, Freedman B (2020). Consumer-led screening for atrial fibrillation using consumer-facing wearables, devices and apps: A survey of health care professionals by AF-SCREEN international collaboration. Eur J Intern Med.

