# Supplementary material for: Automatic Mobile Health Arrhythmia Monitoring for the Detection of Atrial Fibrillation: Prospective Feasibility, Accuracy, and User Experience Study
Source: JMIR Mhealth Uhealth. 2021 Oct 22;9(10):e29933. doi: 10.2196/29933 (PMC8571685; doi:10.2196/29933)
Supplement: Multimedia Appendix 1 [file mhealth_v9i10e29933_app1.pdf]

Supplementary material A
Validation of the Atrial fibrillation detection algorithm

Background of the validation
It can be problematic to distinguish atrial fibrillation from atrial flutter. Both arrythmias are associated with a similar risk of stroke. Therefore, the abbreviation "AF" is used to refer to both atrial flutter and atrial fibrillation in this document.

Materials
Validation of the algorithm for AF detection was performed by using four public datasets (MIT-BIH Arrhythmia Database (Mit-Arr) [1], MIT-BIH Atrial fibrillation database (Mit-AF) [2], Long-Term AF Database (Long-AF) [3], MIT-BIH Normal Sinus Rhythm Database (Normal SR) [4]) freely available for research purposes on PhysioNet [4]. All datasets contain original ECG-signal and reference rhythm annotations. The combination of these datasets contains 173 recordings (duration 30min – 24h) in which 97 measurements contain at least one >5 min AF episode. The dataset contains more than 7700 diagnostic (episode length >30s) and more than 650 >5 min AF episodes. The total duration of measurements is 2656h of which 1147h were AF.

Results
The sensitivity of the algorithm to detect short AF-episodes was estimated as moderate as the algorithm detected 83% of all ≥30sec AF episodes (Supplementary Figure 1). The sensitivity increased significantly in conjunction with the duration of AF; 95% and 98% from >5 min and >15min episodes, respectively.

Supplementary Figure 1: The number of AF-episodes in the whole dataset is presented in the left panel. The sensitivity of the automated algorithm for detecting AF-episodes is presented in the right panel.

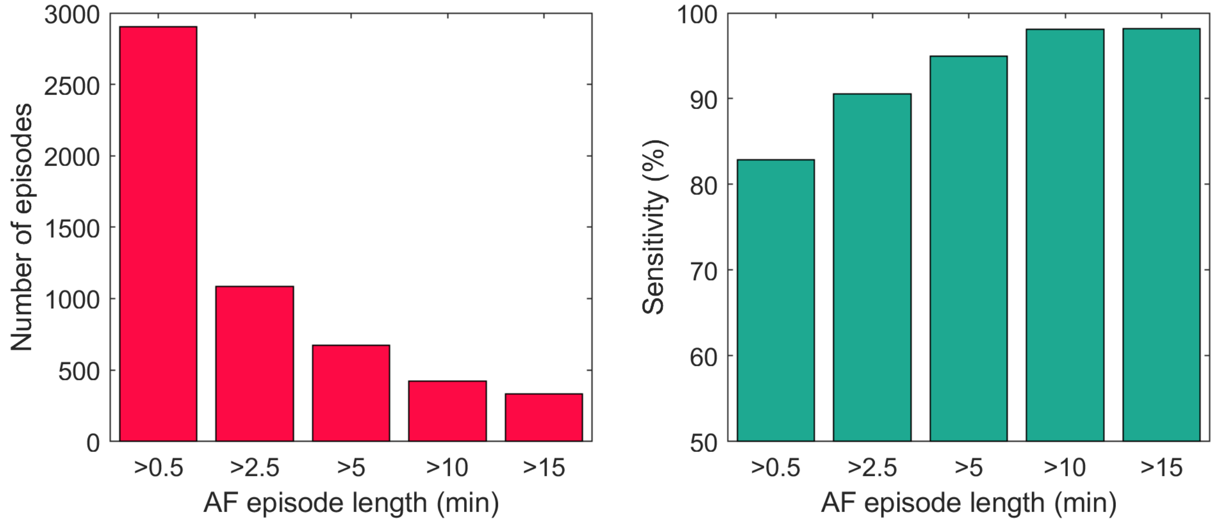

The AF-burden estimate generated by the automatic algorithm was compared to the reference AF-burden (correlation plot of AF-burden is presented in Supplementary Figure 2 left panel). The correlation between the true and the algorithm estimated burden was >0.99 and the burden error was 0.05h ± 0.82 hours (mean ± SD).

Supplementary Figure 2: AF-burden correlation plot between reference burden and AF- burden estimated by the automatic algorithm.

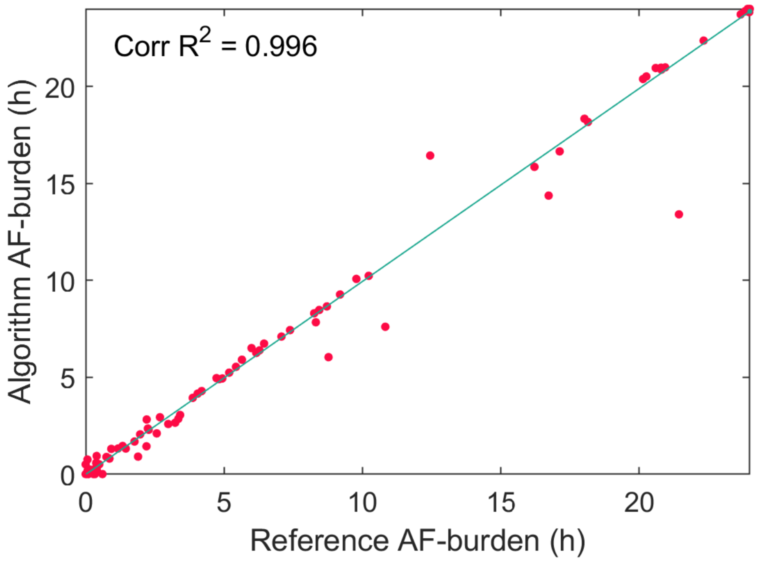

The overall time-based performance measures for the automatic AF detection by the algorithm are presented in Supplementary Table 1. The overall sensitivity for AF detection was high 97.1% with a specificity of 98.4%. The specificity of the algorithm for AF was decreased by the large amount of supraventricular ectopic beats in the AF-datasets, which commonly appear before and after the AF episodes. In addition, the positive predictive value of the algorithm for AF in the Mit-Arr dataset was low, due to the low amount of AF in that dataset. Importantly, the specificity of the algorithm was 100% in the normal sinus rhythm dataset.

Supplementary Table 1: Time based performance measures for individual datasets.

	MIT-AF	Long-AF	Mit-Arr	Normal SR	All
Sensitivity	96.3	97.2	96.2		97.1
Specificity	96.8	97.9	95.4	100	98.4
Pos pred.	95.3	98.2	70.5		97.8
Neg pred.	97.4	96.8	99.5	100	97.8
Accuracy	96.6	97.5	95.5	100	97.8

Supplementary material A: References

1. Moody GB, Mark RG. The impact of the MIT-BIH arrhythmia database. IEEE Eng Med Biol Mag. 2001;20(3):45-50. doi:10.1109/51.932724. PMID: 11446209
2. Moody GB, Mark RG. A new method for detecting atrial fibrillation using R-R intervals. Computers in Cardiology. 1983;10:227-230.
3. Petrutiu S, Sahakian AV, Swiryn S. Abrupt changes in fibrillatory wave characteristics at the termination of paroxysmal atrial fibrillation in humans. Europace. 2007;9(7):466-470. doi:10.1093/europace/eum096. PMID: 17540663
4. Goldberger AL, Amaral LA, Glass L, et al. PhysioBank, PhysioToolkit, and PhysioNet: components of a new research resource for complex physiologic signals. Circulation. 2000;101(23):E215-E220. doi:10.1161/01.cir.101.23.e215. PMID: 10851218
